# Supplementary figures and images for: Recent loss of the Dim2 DNA methyltransferase decreases mutation rate in repeats and changes evolutionary trajectory in a fungal pathogen
Source: PLoS Genet. 2021 Mar 22;17(3):e1009448. doi: 10.1371/journal.pgen.1009448 (PMC8016269; doi:10.1371/journal.pgen.1009448)

A) *dim2*

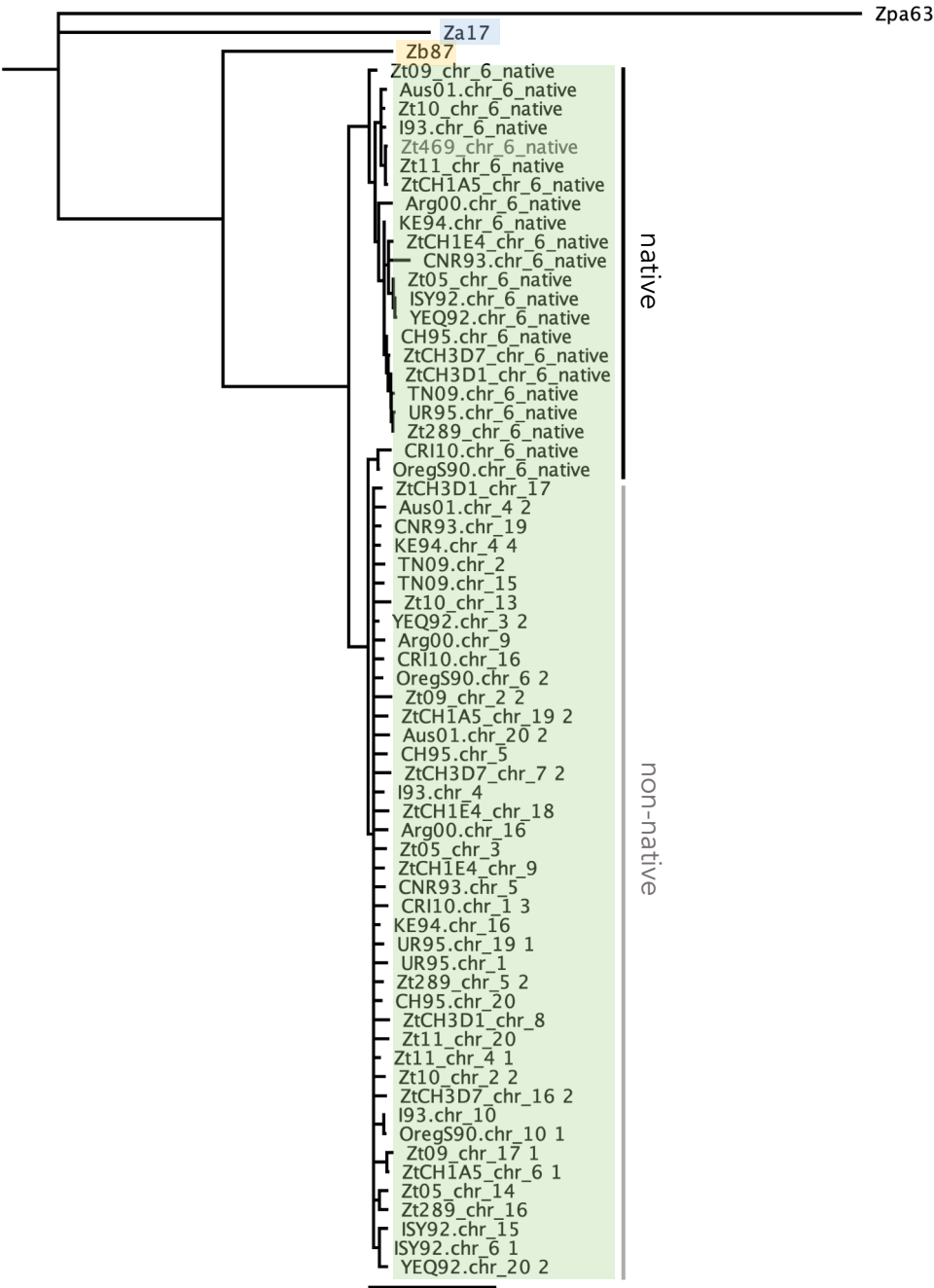

B) *dnmt5*

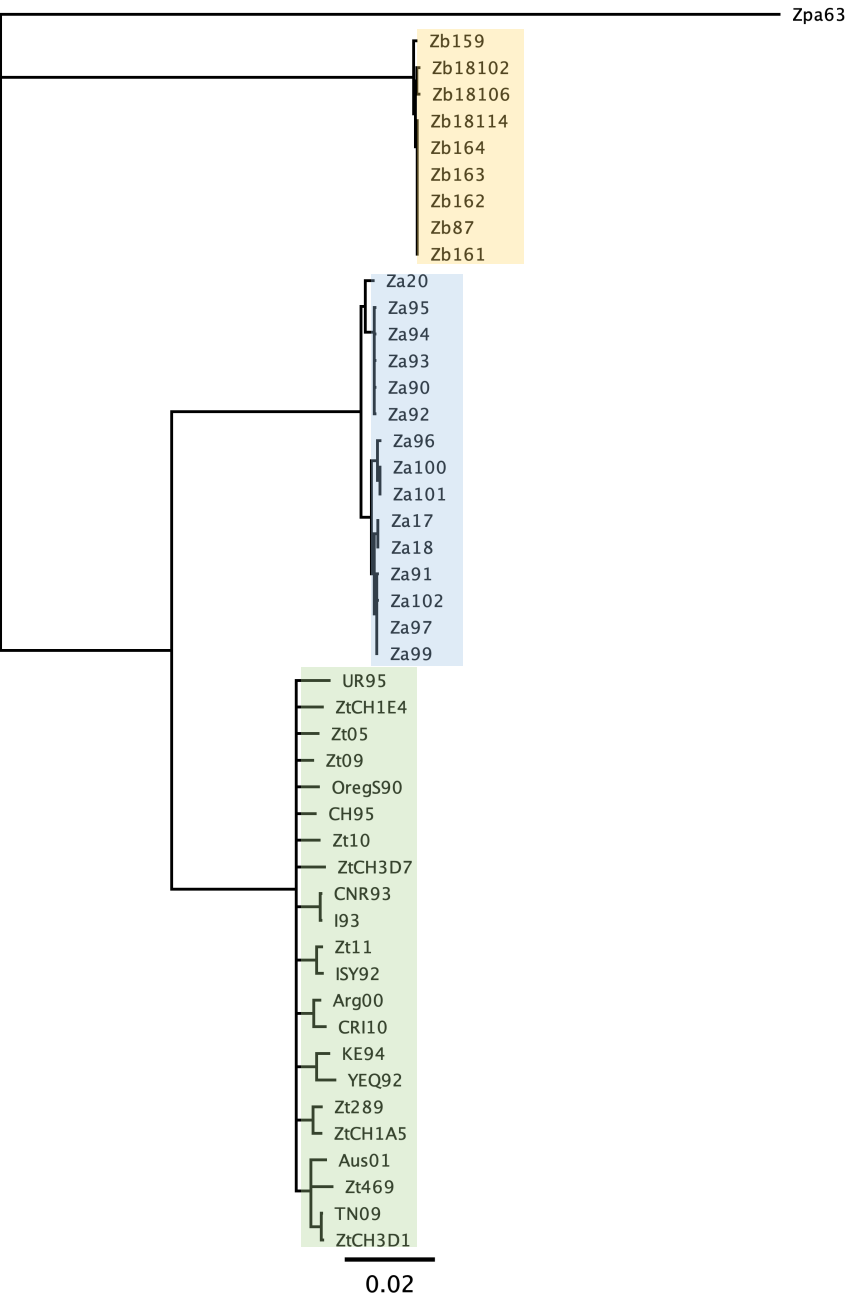

C) *rid*

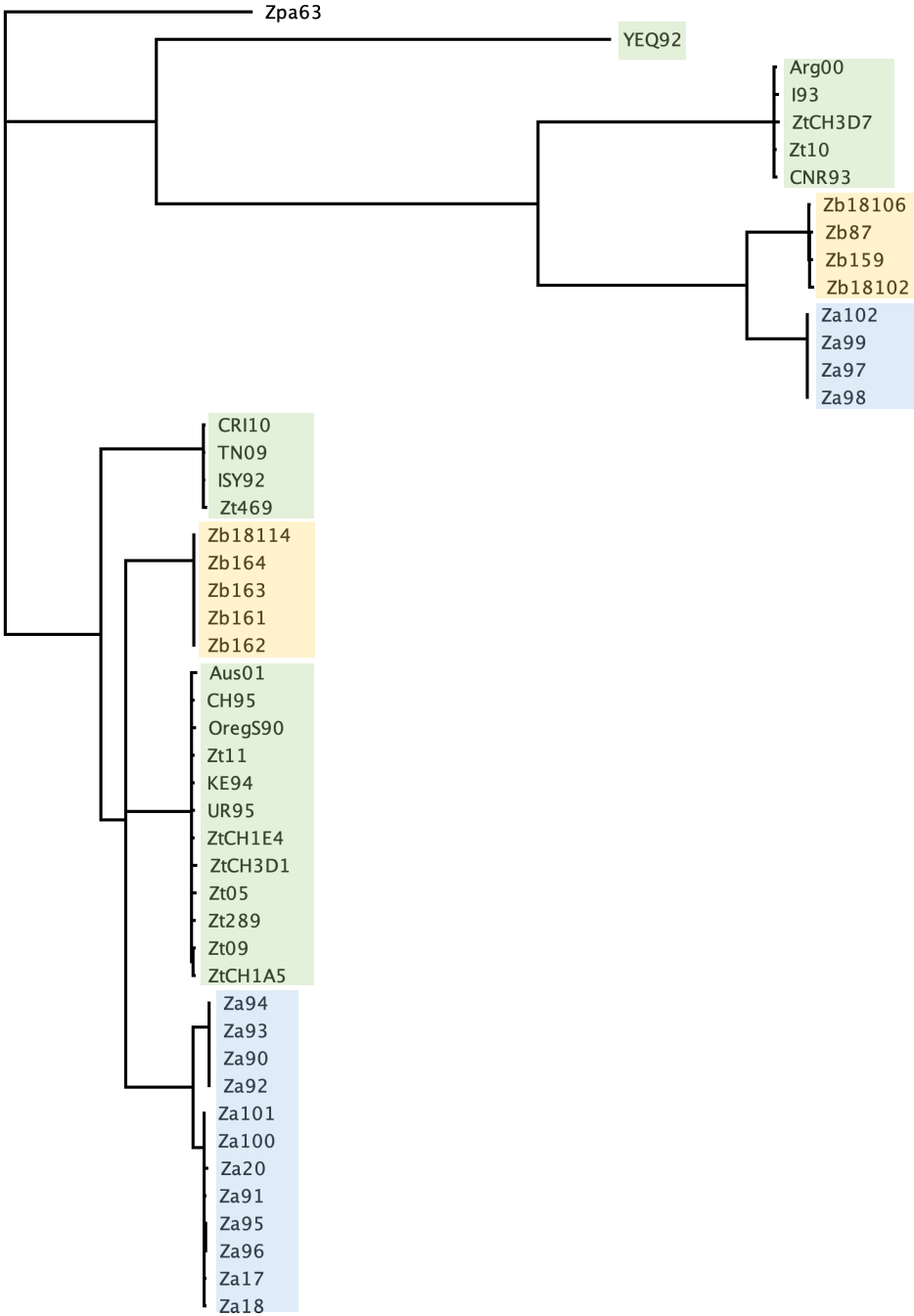

0.06

Supplement: S1 Fig — (A) Phylogenetic tree based on alignments of “deRIPed” dim2 alleles to the native, functional gene of Zt469. The Z. tritici dim2 is distinct from the Z. brevis and Z. ardabiliae gene. The native Z. tritici copies, except for OregS90 and CRI10, and non-native copies form distinct clusters. Shown are two representative “deRIPed” non-native copies per isolate. (B) Dnmt5 is present in all analyzed genomes and shows relatively little inter- or intraspecies diversity. (C) The rid gene shows an exceptionally high inter- and intraspecies diversity with three highly distinct alleles present among genomes of Z. tritici, Z. ardabiliae and Z. brevis. Green background indicates Z. tritici, blue Z. ardabiliae, yellow Z. brevis. Z. passerinii is used as an outgroup. (PDF) [file pgen.1009448.s001.pdf]

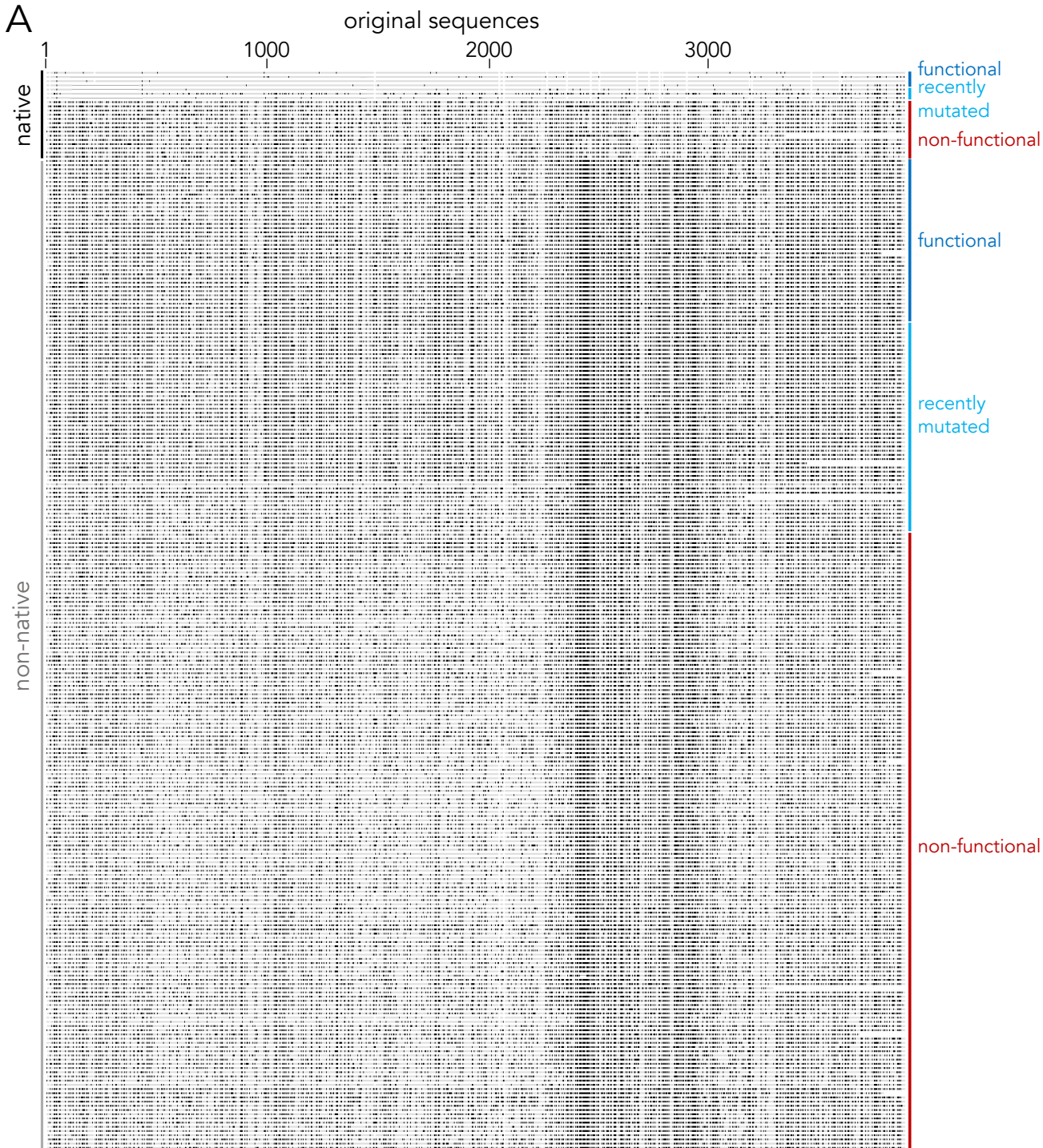

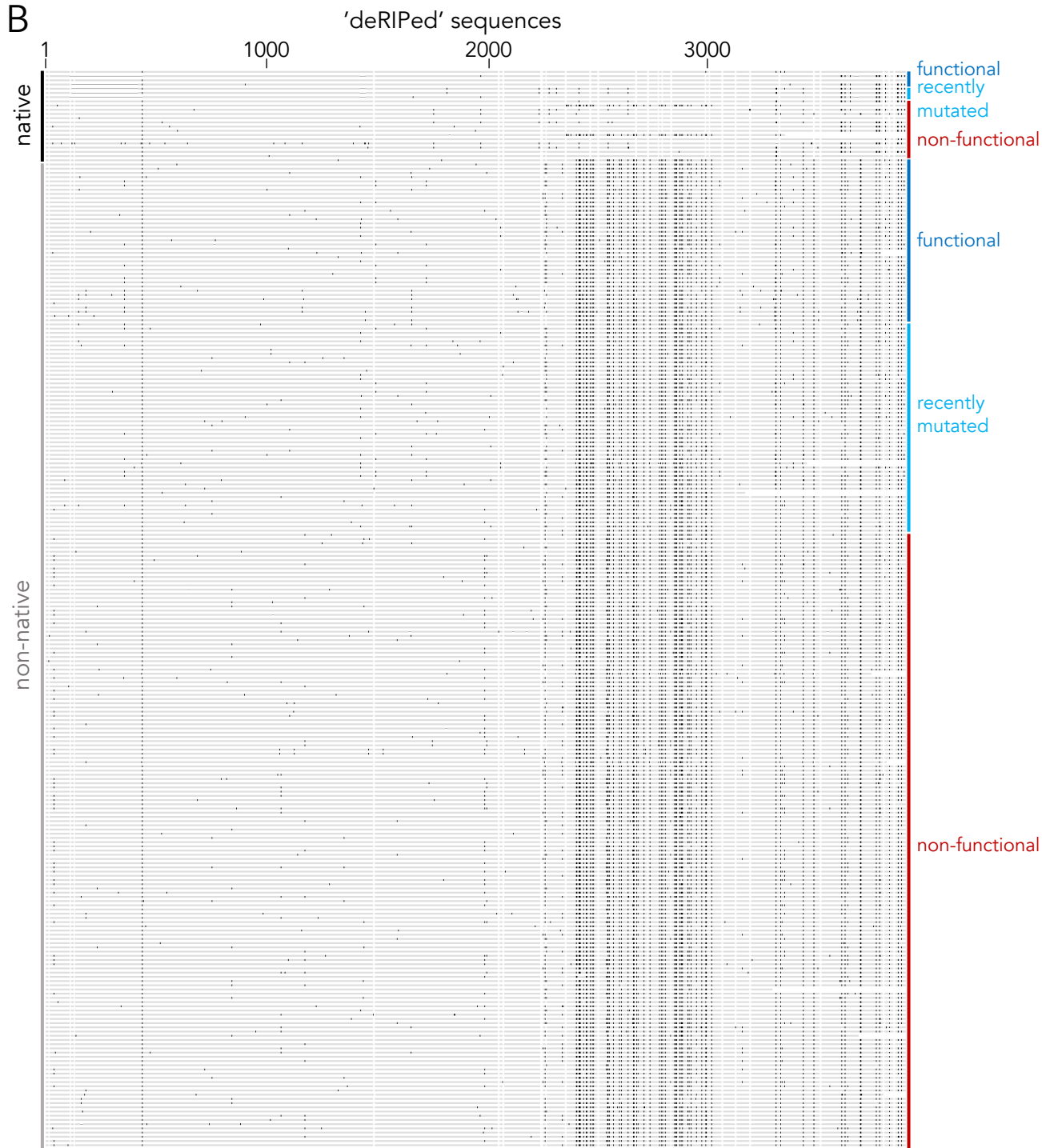

Supplement: S2 Fig — Alignment of original (A) and ‘deRIPed’ (B) full-length (> 3000 bp) dim2 alleles compared to the functional allele of isolate Zt469. All native copies, (functional and non-functional, except for OregS90 and CRI10) lack mutations in the DNA methyltransferase domain (position ~2,300–3,500) that are present in all non-native copies suggesting that the additional copies did not emerge from amplification of the native dim2. Black lines indicate differences to the reference. (PDF) [file pgen.1009448.s002.pdf]

A

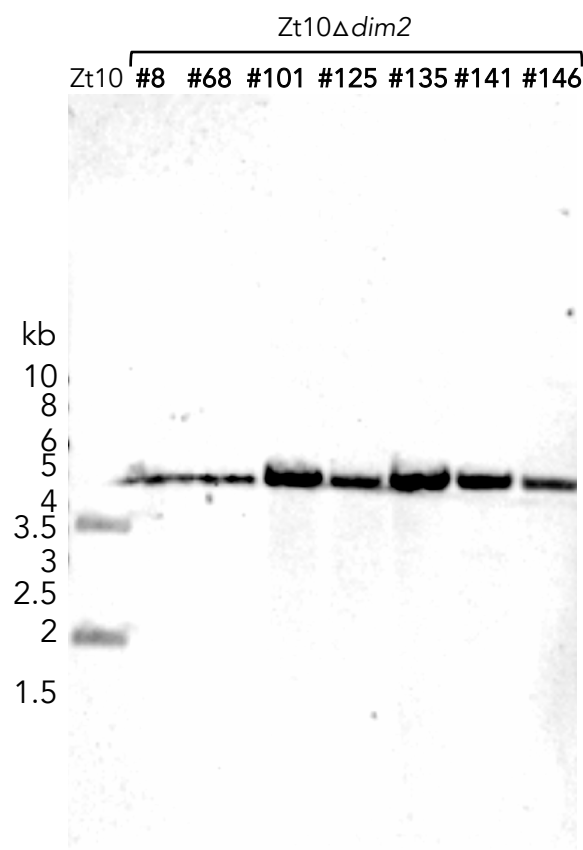

Enzyme *Sall*  
Zt10 2027 bp  
Zt10ΔZtdim2 3572 bp  
Zt10ΔZtdim2 4815 bp

B

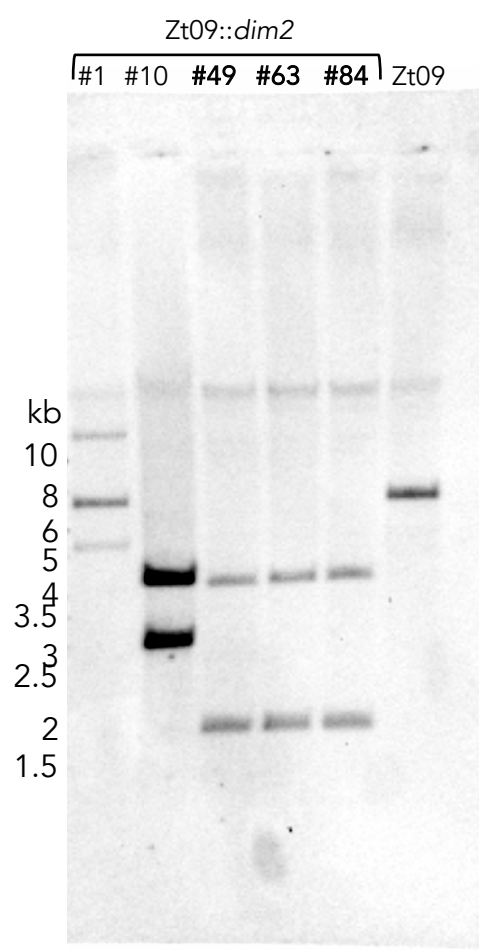

Enzyme *NcoI*  
Zt09 7122 bp  
Zt09::Ztdim2 1825 bp  
Zt09::Ztdim2 4176 bp

Supplement: S4 Fig — Three positive transformants (#49, #63 and #84) were found amongst the Zt09::dim2 candidates, whereas all seven candidates for Zt10Δdim2 were verified (correct transformants are highlighted in bold). (PDF) [file pgen.1009448.s004.pdf]

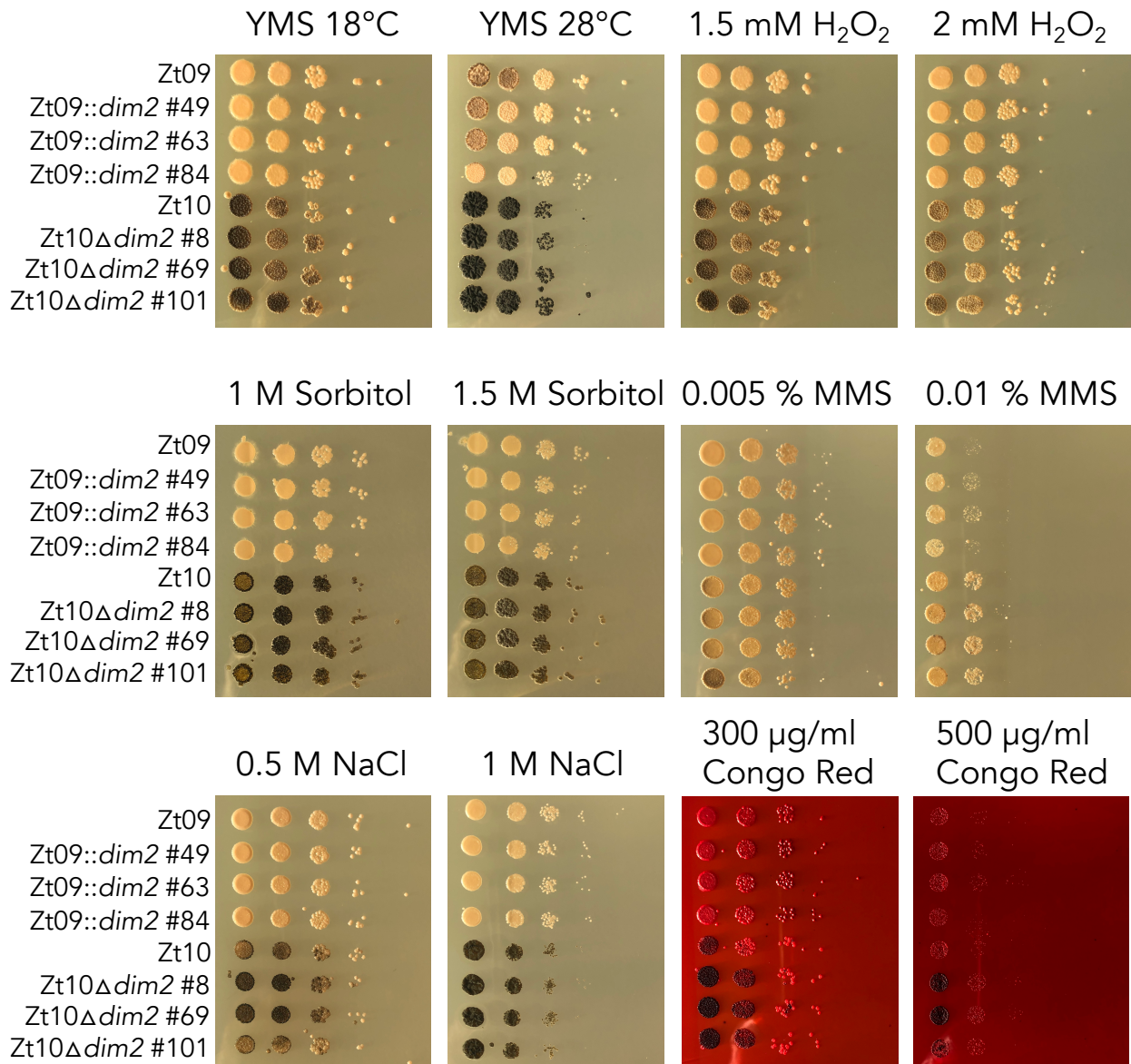

Supplement: S5 Fig — We compared growth phenotypes under different in vitro conditions including temperature, osmotic, oxidative, genotoxic and cell wall stress. We spotted spore dilutions of each reference isolate and three independent dim2 mutant transformants on each plate. We did not detect any noticeable differences in growth between reference and mutant strains. Previously described differences between the Z. tritici isolates Zt09 and Zt10 were observed [35]. (PDF) [file pgen.1009448.s005.pdf]

A

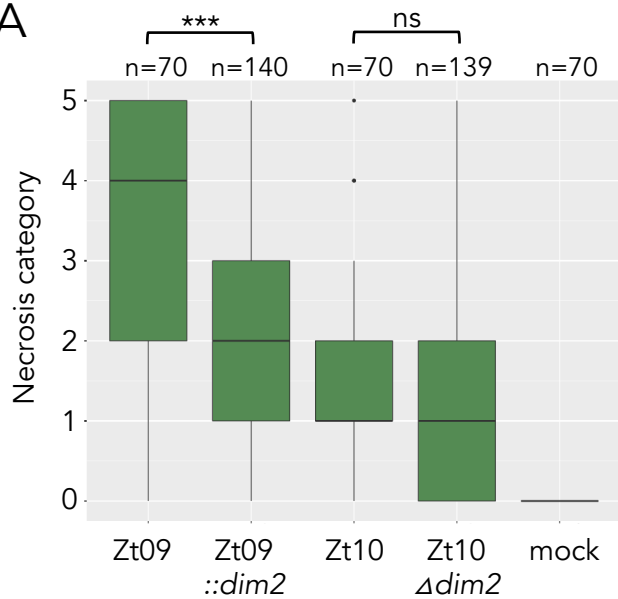

B

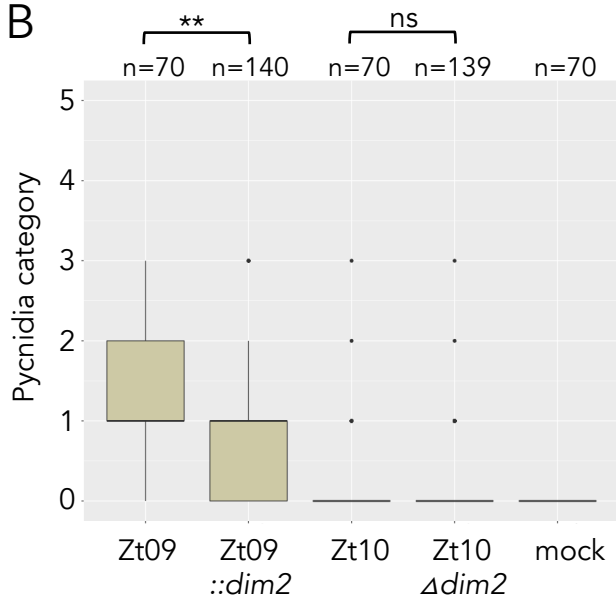

Supplement: S6 Fig — (A) Zt09::dim2 strains show significantly less necrotic lesions compared to Zt09 (*** Wilcoxon rank-sum test, p-value = 2.178 x 10^−7^) while there is no significant difference in the quantities of necrotic lesions caused by Zt10 and Zt10Δdim2 strains. (B) Coverage with pycnidia is significantly reduced between Zt09 and Zt09::dim2 (** Wilcoxon rank-sum test, p-value = 0.003301) but not between Zt10 and Zt10Δdim2 strains. Categories for necrotic lesion and pycnidia coverage: 0 = 0%, 1 = 1–20%, 2 = 21–40%, 3 = 41–60%, 4 = 61–80%, 5 = 81–100%. (PDF) [file pgen.1009448.s006.pdf]

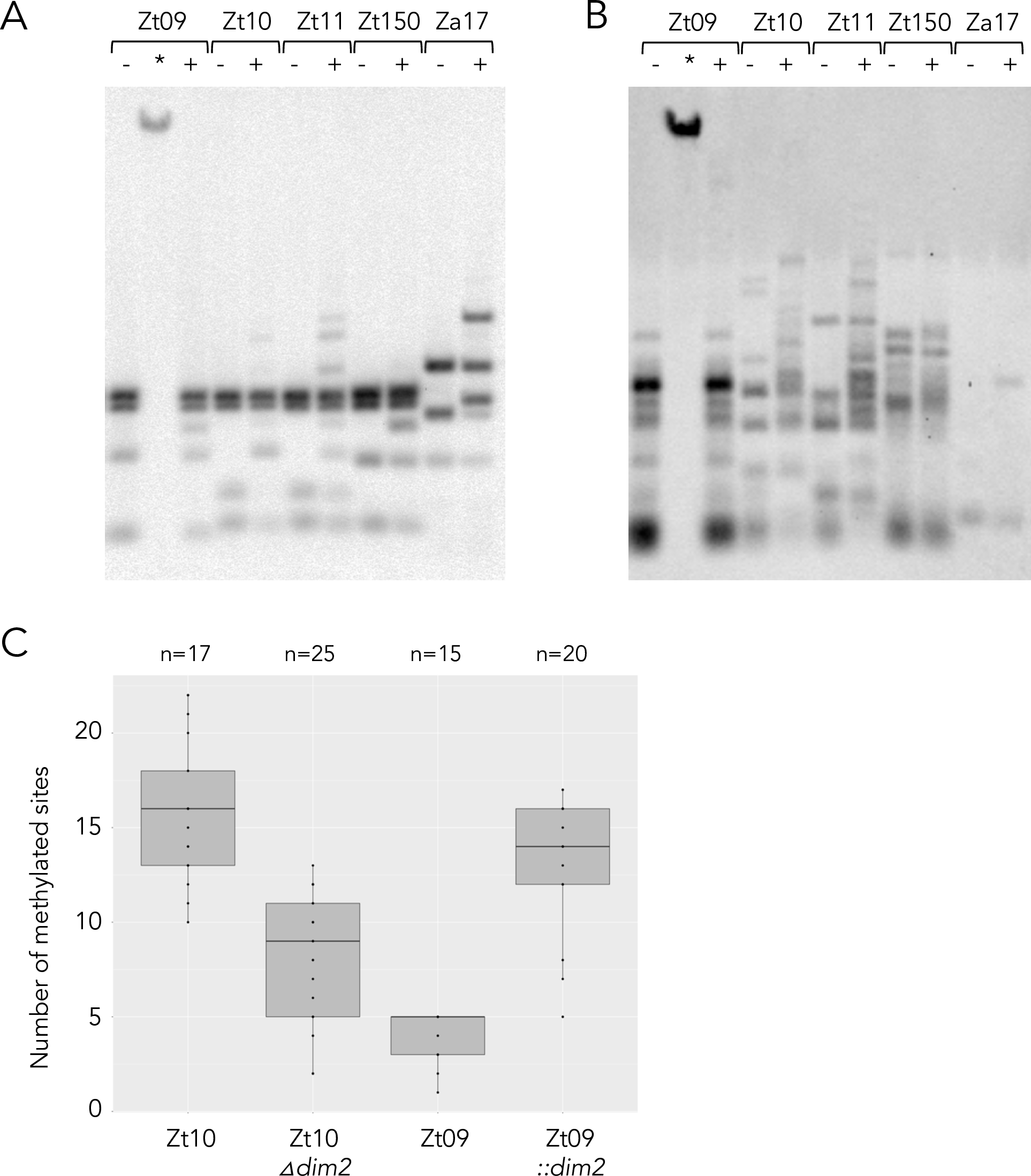

Supplement: S7 Fig — Restriction enzyme analysis followed by Southern blots using the rDNA spacer (A) or a retrotransposon RIL2 (B) as probe. In addition to the cytosine methylation sensitive (BfuCI, +) or insensitive (DpnII, -) enzymes, we used DpnI (*) to test for the presence of adenine methylation. Zt09 and Zt150 contain a non-functional dim2, whereas the Iranian strains Zt10, Zt11 and the Z. ardabiliae strain Za17 have a functional copy. In all strains with a functional dim2 we see a clear difference between the restrictions with BfuCI and DpnII, indicating the presence of DNA methylation. In Zt09 and Zt150 this difference is not detectable except for one band that is not restricted in both strains in the rDNA blot. The genomic DNA of Zt09 treated with DpnI is not digested suggesting absence of 6mA methylation in these regions. (C) Confirmation of bisulfite sequencing by an independent bisulfite treatment followed by PCR of target loci and Sanger sequencing. Two target loci (repeated region that showed 5mC signals in all isolates) were chosen per isolate, shown are the number of detected 5mC sites in these loci (size of loci ~ 180–260 bp). N is the number of cloned PCR products that were sequenced. Based on these data we can confirm the presence of 5mC methylation in all isolates and a lower frequency in absence of a functional dim2. (TIF) [file pgen.1009448.s007.tif]

**A**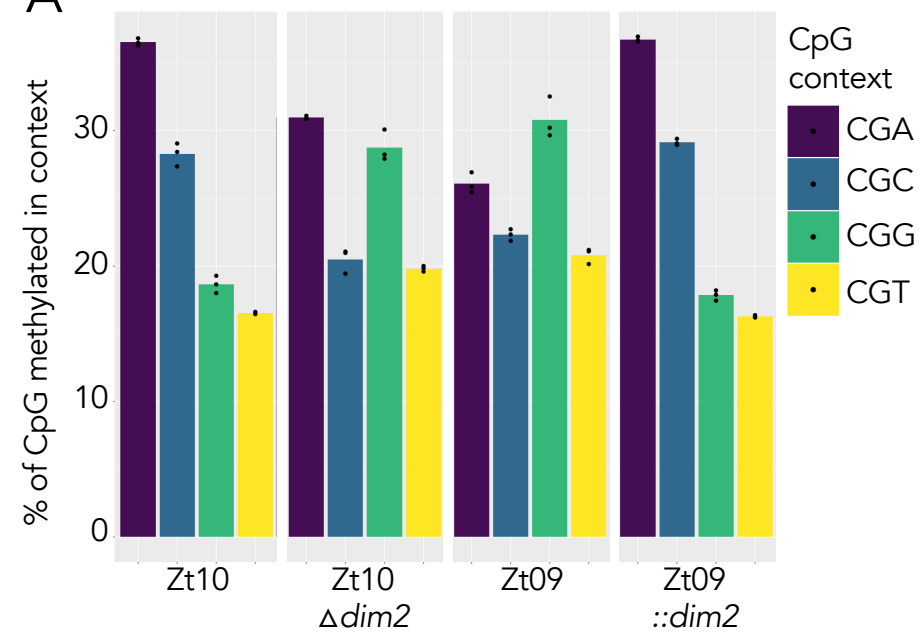**B**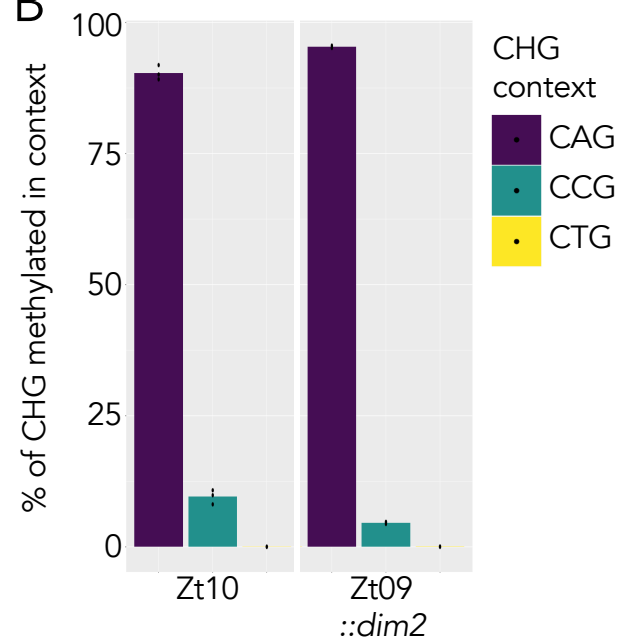**C**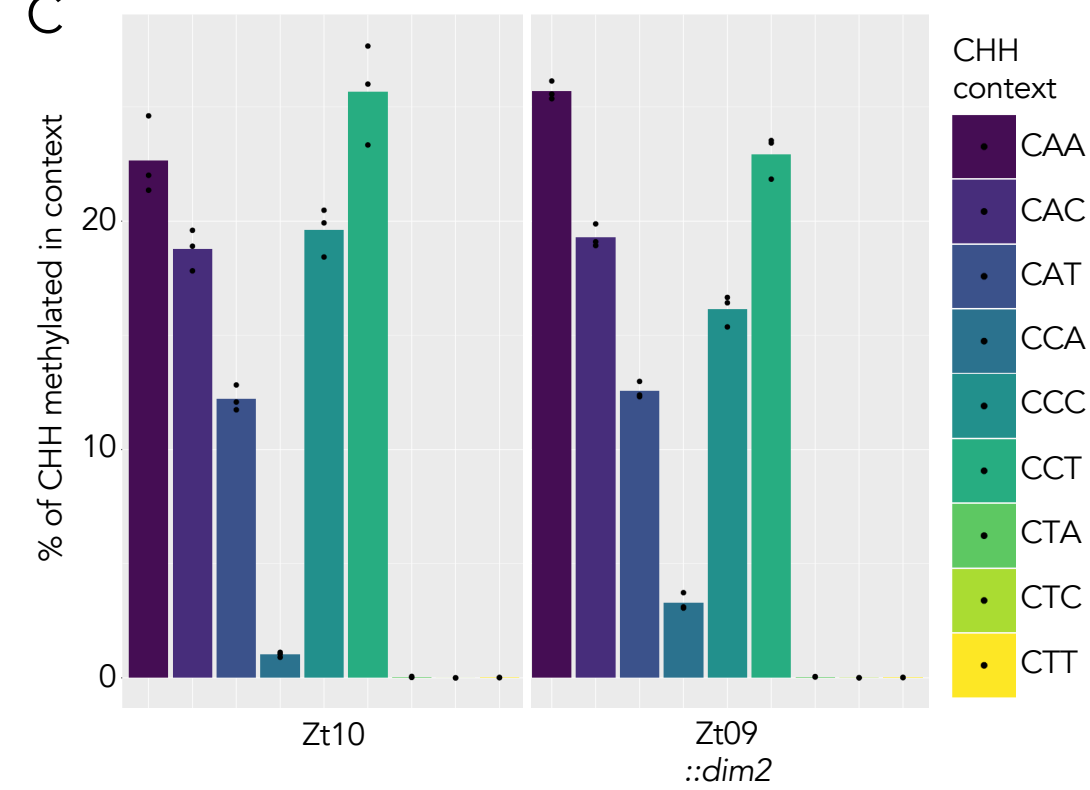

Supplement: S8 Fig — (A) CGA and CGC sites are preferred in CG contexts in presence of dim2, while CGA and CGG are slightly preferred targets in absence of dim2. (B) Among CHG sites, CAG sites are the predominant target sites of 5mC. For CHG and CHH contexts only data for Zt10 and Zt09::dim2 are shown, as there is no detectable methylation outside of CG contexts in absence of a functional dim2. (C) In CHH contexts, CA sites followed by CC sites have the highest methylation frequency. CT sites are almost completely devoid of 5mC. (PDF) [file pgen.1009448.s008.pdf]

A

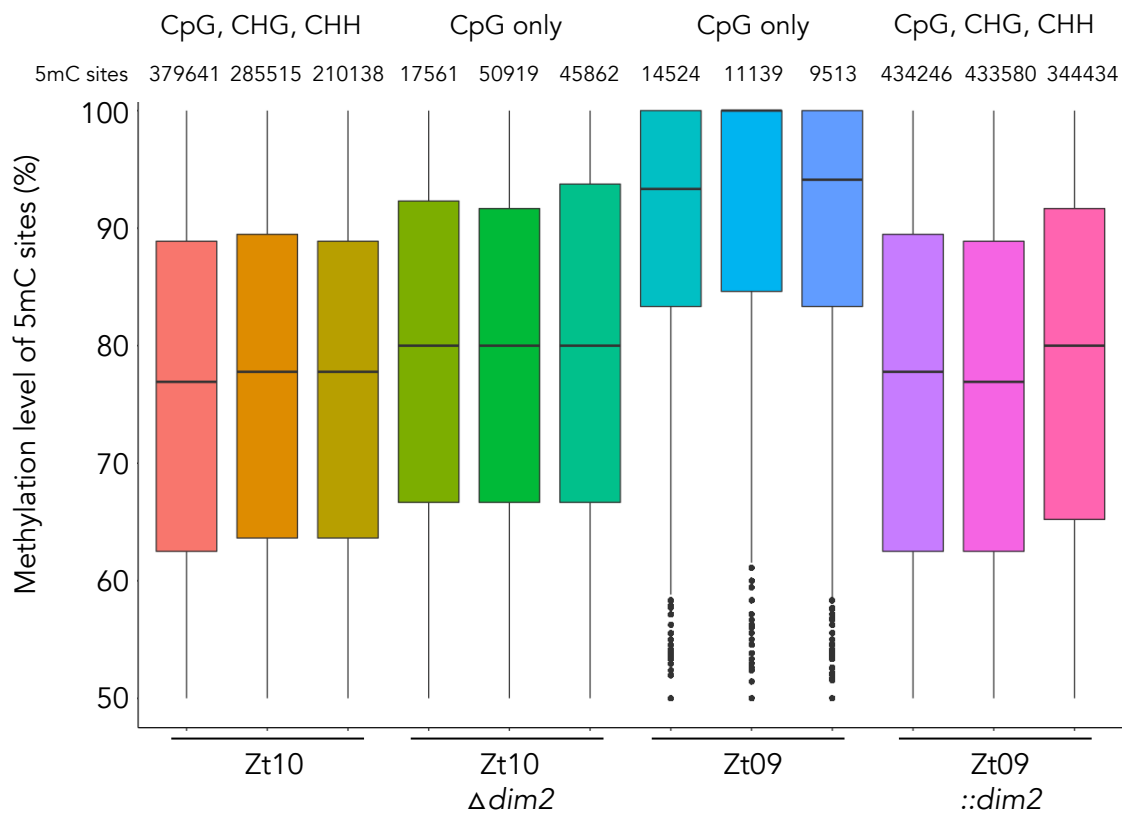

B

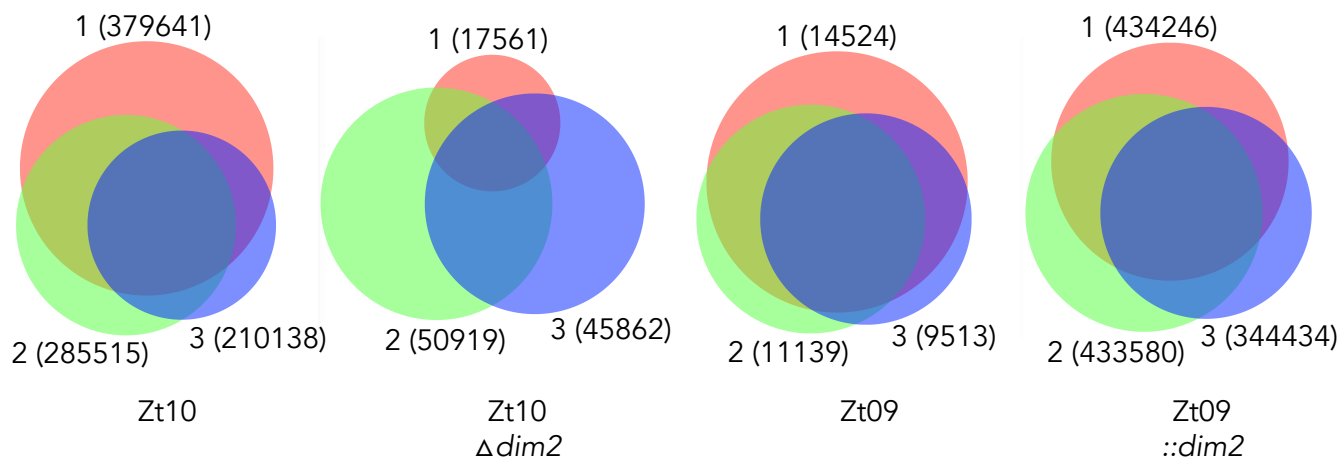

Supplement: S9 Fig — (A) Methylation levels of 5mC sites (5mC reads/site) in the different mutants and replicates. Methylation levels do not drop below 50% as this was the threshold to call 5mC sites. Methylation levels are highest in Zt09, where only few CpG sites are maintained by Dnmt5. All other strains have methylation levels mostly between 70–80% indicating more heterogenity of 5mC sites within the cell populations. (B) Overlap of detected 5mC sites between replicates of the same strain. Zt10 and Zt09 replicates originate from three independent inoculations while the mutant replicates represent independent transformants. The largest differences in terms of 5mC site overlap can be observed in the Zt10Δdim2 mutant. A likely explanation is that 5mC sites are in the process of being lost in absence of dim2 and that different sites get lost at different times in the independent transformants. (PDF) [file pgen.1009448.s009.pdf]

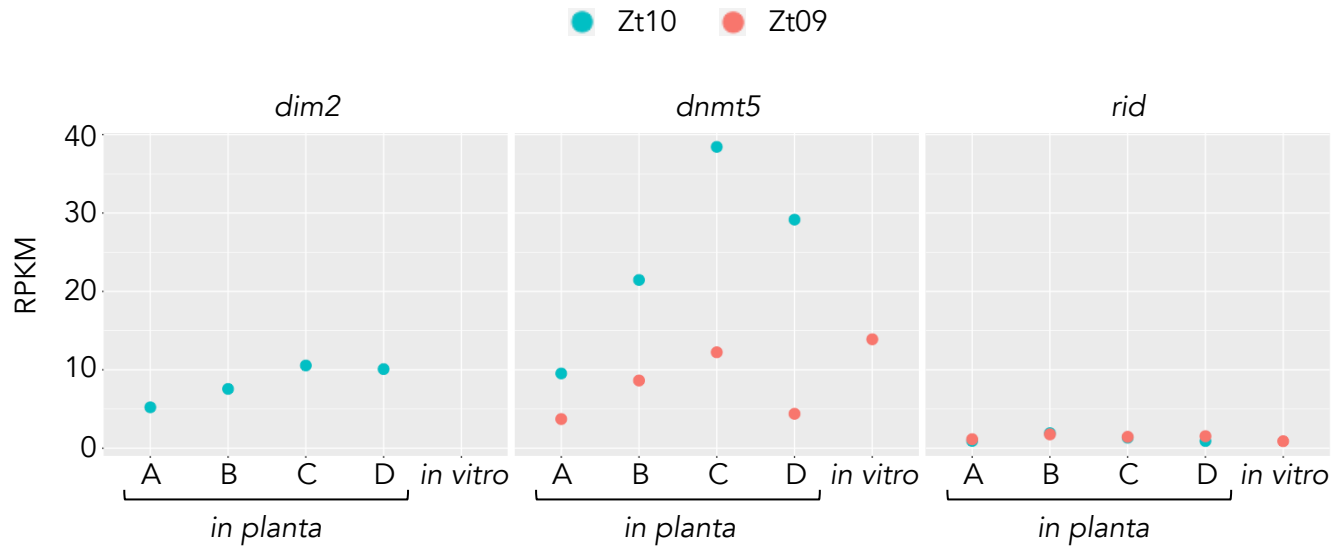

Supplement: S10 Fig — In vitro data is only available for Zt09. Expression during infection was monitored in specific stages: A–infection establishment, B–biotrophic growth, C–transition from biotrophic to necrotrophic growth, D–necrotrophic colonization [35]. (PDF) [file pgen.1009448.s010.pdf]
