# Supplementary material for: Recent loss of the Dim2 DNA methyltransferase decreases mutation rate in repeats and changes evolutionary trajectory in a fungal pathogen
Source: PLoS Genet. 2021 Mar 22;17(3):e1009448. doi: 10.1371/journal.pgen.1009448 (PMC8016269; doi:10.1371/journal.pgen.1009448)
Supplement: S3 Fig — (A) Cartoon of complete protein coding sequences. The conserved domains detected by homology searches are indicated above the alignment (BAH, Bromo-Adjacent Homology domain). The top four alleles (Zt467, Zt468, Zt469, Zt471) were identified in strains isolated from Aegilops, all others were isolated from wheat. Alleles from CRI10 and Zt09 are non-functional and were “de-RIPed” as described in the main text. Sequences of alleles from Zt289 and TN09 suggest the presence of several potentially functional dim2 alleles in natural populations of Z. tritici. Color scheme according to rasmol (http://www.pdg.cnb.uam.es/cursos/Barcelona2002/pages/Farmac/CMU_course/ProtG/ShapelyColors.html). (B) Complete sequence alignment of all alleles shown in the cartoon. All SNVs and indels that lead to non-synonymous changes are indicated; there are many more synonymous than non-synonymous mutations in the various alleles. The conserved ten canonical motifs found in most DNMTases are underlined and indicated with Roman numerals. Motif I is the SAM (Adomet)-binding domain, motif IV includes the catalytic site with the invariant Pro-Cys site. Motif VIII is the most variable in Zymoseptoria dim2 alleles–this region is considered a poorly conserved targeting domain in bacterial DNMTases. Amino acids indicated in bold red show variations likely caused by the deRIP procedure (i.e., C or G were introduced when T or A would have retained the conserved amino acid, e.g. TCN->CCN, Ser, conserved, to Pro, de-RIP, or CAR->CGR, Gln, conserved, to Arg, de-RIP). Amino acids shown in blue suggest the presence of two or more different alleles in the Z. tritici population; most of these differences occur at the non-conserved C-terminus. Mutation of the predominant TAG/A stop codon to CAG/A (Q) generated a short addition of nine amino acids in two alleles. In addition to the known Dim2 domains, the two BAH (aa 494–601 and 637–748) and DNMT catalytic domain (aa 694–1171), we predict several other motifs that [file pgen.1009448.s003.pdf]

A

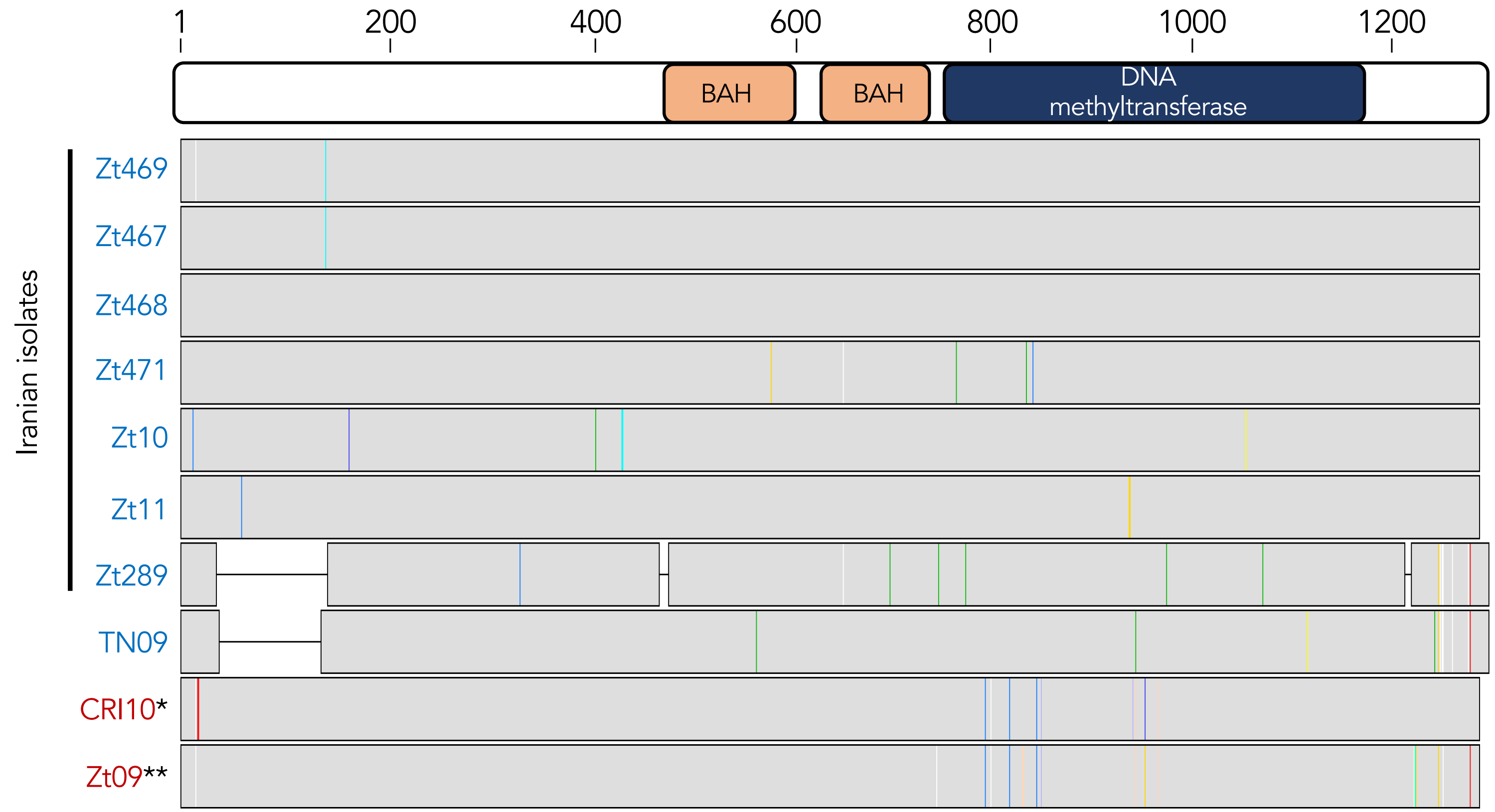

*de-RIPed native allele

**de-RIPed transversion allele (chr 5)

B

```
Zt469     1 MKRRISGDTIVIEDDGDNIVVGPTSRPSKKPRLQNGQLQMDILRAGASLSNAIVISPAASEQDRGESKQQEEDGAQRAVQYEMESGEEQTSQTVGDREVEDQGPLRTPRQRRLRTALTPP 120
Zt467     1 ........................................................................................................................
Zt468     1 ........................................................................................................................
Zt471     1 ........................................................................................................................
Zt10      1 ............K...........................................................................................................
Zt11      1 ............................................................K...........................................................
Zt289     1 ....................................------------------------------------------------------------------------------------
TN09      1 .......................................---------------------------------------------------------------------------------
CRI10     1 ...............G.D......................................................................................................
Zt09      1 ...............G........................................................................................................
            ************ ** * ******************

Zt469   121 ATETEPGDEPAARAEPGATARSGNRRQLRYLSHISIAGPAFPRSAYHGYKPPLQPVNEVEASLDLSAGFFAAAKSAGTETPECATFKLDQFSVYMADGRRASDLVTLEKLRQSNYAEFCF 240
Zt467   121 .......................N................................................................................................
Zt468   121 .......................K................................................................................................
Zt471   121 .......................K................................................................................................
Zt10    121 .......................K......................Y.........................................................................
Zt11    121 .......................K................................................................................................
Zt289    37 -------------------------...............................................................................................
TN09     40 ------------------.....K................................................................................................
CRI10   121 .......................K................................................................................................
Zt09    121 .......................K................................................................................................
                                     ********************* *************************************************************************

                                                N-terminal RFD-like motif in Dim2
Zt469   241 DGILSAGNEKRYVQGVRFEILAISGYGDLDFTDFAGSICIQSKLQSKASALFGHWYELGTPAPEYRRFYEPFLWLARFGKHFVDYLLSNQQVTLGHFRRRFYQWLTGLYGDDTSFNQWLA 360
Zt467   241 ........................................................................................................................
Zt468   241 ........................................................................................................................
Zt471   241 ........................................................................................................................
Zt10    241 ........................................................................................................................
Zt11    241 ........................................................................................................................
Zt289   132 ..............................................................................................R.........................
TN09    142 ........................................................................................................................
CRI10   241 ........................................................................................................................
Zt09    241 ........................................................................................................................
            ********************************************************************************************** *************************

                                                                 short conserved motif                          NLS
Zt469   361 ECKLKDFRTTIAANVGFLWKECWDIDDKESGLCRQPIWGEVDPYNLTAIPAQPNKELRTIVTPFVYQCFRHMYFGDQLEERTITDRNVLKKSDELKARLRLTPRRASPSAPPMQTLTPRS 480
Zt467   361 ........................................................................................................................
Zt468   361 ........................................................................................................................
Zt471   361 ........................................................................................................................
Zt10    361 .................................................L.........................N............................................
Zt11    361 ........................................................................................................................
Zt289   252 ................................................................................................................--------
TN09    262 ........................................................................................................................
CRI10   361 ........................................................................................................................
Zt09    361 ........................................................................................................................
            ************************************************* ************************* ************************************
```

```
                                                                           BAH domain 1
Zt469  481 MSRSESQEGNKAVTVNTGDVVCVAPDVSTKWGIKAHAWYAYVQNVHTRKDRTSLDVLWLYEPKDTTLGKAYYPFANELFLSDNCSCGTSALDLDCVLDKADVCWYAKDPYQQGGHFVRQK 600
Zt467  481 ........................................................................................................................
Zt468  481 ........................................................................................................................
Zt471  481 ......................................................................................................S.................
Zt10   481 ........................................................................................................................
Zt11   481 ........................................................................................................................
Zt289  364 -.......................................................................................................................
TN09   382 .......................................................................................I................................
CRI10  481 ........................................................................................................................
Zt09   481 ........................................................................................................................
            ************************************************************************************** **************.*****************

                                                                                                  BAH domain 2
Zt469  601 FRTVHEQDTYDFVAIQASDFRCQCKHDVPIFDECRSKYAIGDTVLVREWNDDLDEDRLQPAQIIEFDTQKRRVVLRQFLRKSEQQTGAKARPNELVLTDVVFDKACDKIIRKCHVRCFRQ 720
Zt467  601 ........................................................................................................................
Zt468  601 ........................................................................................................................
Zt471  601 .....................................................G..................................................................
Zt10   601 ........................................................................................................................
Zt11   601 ........................................................................................................................
Zt289  483 .....................................................G.............................................I....................
TN09   502 ........................................................................................................................
CRI10  601 ........................................................................................................................
Zt09   601 ........................................................................................................................
           ***************************************************** *********************************************.********************

                                                                      motif I (SAM-binding)
Zt469  721 DQIDSGDIVTPYDRDGTGDFFYLAEDLSAARDGFPPMKEGWNPNMHHGEKMVGMSLFCGGGTFDRGLEEAGIAKFKYAVDWNEHALHSHRANVRRPDQMEYFLGSVNDLLARAMAGDPKP 840
Zt467  721 ........................................................................................................................
Zt468  721 ........................................................................................................................
Zt471  721 ............................................V.....................................................................I......
Zt10   721 ........................................................................................................................
Zt11   721 ........................................................................................................................
Zt289  603 ...........................L.........................I..................................................................
TN09   622 ........................................................................................................................
CRI10  721 .........................................................................R....A..................R......................
Zt09   721 .........................A...............................................R....A..................R............P.........
           ************************* * ****************.********.*******************.**** ******************.************ **.******

              motif IV (catalytic site)      motif V         motif VI                       motif VII              motif VIII
Zt469  841 NIAQPGDIQILAGGSPCPGFSMLQLFKLSEQSKQNASLVASIVSFVDFYVPRYFLLENVVTMTAGMGPNKDQNVFSQIVAALVALGYQVQQFLGDAWSYGSSQQRSRVFIVASAPGLPPL 960
Zt467  841 ........................................................................................................................
Zt468  841 ........................................................................................................................
Zt471  841 K.......................................................................................................................
Zt10   841 ........................................................................................................................
Zt11   841 ...............................................................................................T........................
Zt289  723 ........................................................................................................................
TN09   742 .....................................................................................................L..................
CRI10  841 ...R....H.......................................................................................H....P........F.........
Zt09   841 ...R....H............................................................................................P........S.........
           **.****.**************************************************************************************.** ** ******** *********
```

```
Zt469  961 SPIAHSHSHPNMSFRSRALGLSSNGLPFGSRRDEFTAFPHVTPAQACADLPDIGDAQPQLCPQFPDHRTMSLESATNRTRMAAVPTRPHGVGLMQAKLLGLLRGEPSTFCDNVNATRRQE 1080
Zt467  961 ........................................................................................................................
Zt468  961 ........................................................................................................................
Zt471  961 ........................................................................................................................
Zt10   961 ..........................................................................................M.............................
Zt11   961 ........................................................................................................................
Zt289  843 ...........I..............................................................................................L.............
TN09   862 ........................................................................................................................
CRI10  961 ...P....................................................................................................................
Zt09   961 ...P....................................................................................................................
           *** *******.******************************************************************************.*************** *************

                                                     motif IX                        motif X
Zt469 1081 NSRMFSRMFPNGLFPTIVTKTAIGDGINGRFMHWSQNRIWTVMEARRAQGYQDWEVLIGSPAQQHKIVGNSVDRKVALVLGLSLRESWEQARSQSLFPRASEIRDEHVKEEMDTDITAEA 1200
Zt467 1081 ............................................................................................................K...D.......
Zt468 1081 ............................................................................................................E...G.......
Zt471 1081 ............................................................................................................E...G.......
Zt10  1081 ............................................................................................................K...D.......
Zt11  1081 ............................................................................................................K...D.......
Zt289  963 ............................................................................................................E...G.......
TN09   982 ..............................C.............................................................................E...G.......
CRI10 1081 ............................................................................................................K...D.......
Zt09  1081 ............................................................................................................E...G.......
           ****************************** ***************************************************************************** *** *******

Zt469 1201 SQPATPTKDEDVEIEETASSVATGGALEEEEQSRAPMAPLTPQVDPSARLNIEELDNRTAKMLLFMPEEVGNVNGLEGGEF          1281
Zt467 1201 .......................................L....D........E...............V.N.........          1281
Zt468 1201 .......................................L....D........E...............V.N.........          1281
Zt471 1201 .......................................L....D........E...............V.N.........          1281
Zt10  1201 .......................................L....D........E...............V.N.........          1281
Zt11  1201 .......................................L....D........E...............V.N.........          1281
Zt289 1083 .......------..........................S.A.AG........G...............A.D.........QESLESLRA 1166
TN09  1102 ....................................I..S.A.AG........G...............A.D.........QESLESLRA 1191
CRI10 1201 .......................................L....D........E...............V.N.........          1281
Zt09  1201 ...............GNS.....................S....G........E...............V.D.........          1281
           *******      ** ..******************.** * *  ******** *************** * *********
```
