# Supplementary material for: Recent loss of the Dim2 DNA methyltransferase decreases mutation rate in repeats and changes evolutionary trajectory in a fungal pathogen
Source: PLoS Genet. 2021 Mar 22;17(3):e1009448. doi: 10.1371/journal.pgen.1009448 (PMC8016269; doi:10.1371/journal.pgen.1009448)
Supplement: S11 Fig — Protein sequences were analyzed with SMART [95], NCBI Blast and InterProScan [96]. (PDF) [file pgen.1009448.s011.pdf]

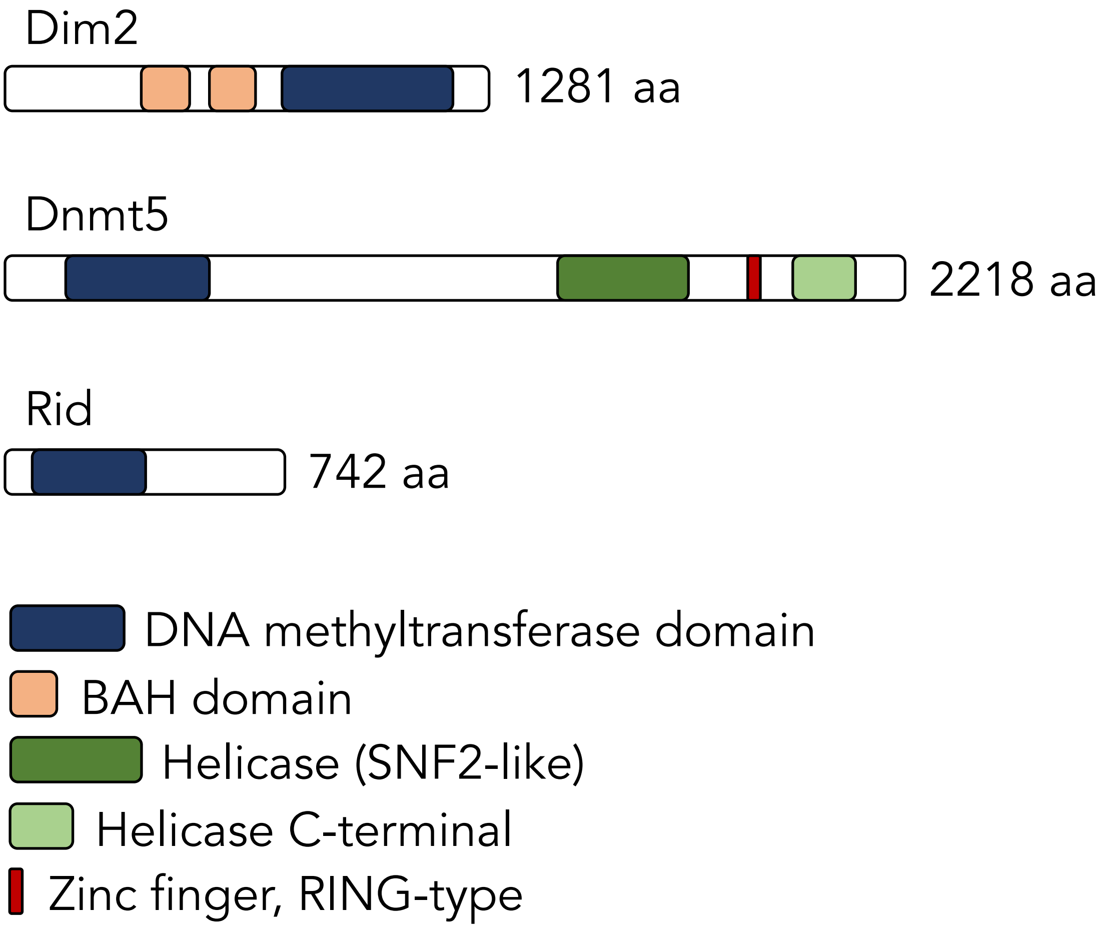
Dim2
1281 aa
Dnmt5
2218 aa
Rid
742 aa
DNA methyltransferase domain
BAH domain
Helicase (SNF2-like)
Helicase C-terminal
Zinc finger, RING-type
