# Supplementary material for: Recent loss of the Dim2 DNA methyltransferase decreases mutation rate in repeats and changes evolutionary trajectory in a fungal pathogen
Source: PLoS Genet. 2021 Mar 22;17(3):e1009448. doi: 10.1371/journal.pgen.1009448 (PMC8016269; doi:10.1371/journal.pgen.1009448)
Supplement: Attachment — Submitted filename: 012421_Responses to Reviewers.docx [file pgen.1009448.s022.docx]

The manuscript received most enthusiastic and supportive feedback from all reviewers all of whom unanimously agreed that the study presents major scientific advancement in area of fungal genome evolution and DNA methylation. We invite you to revise your manuscript addressing all of the reviewer’s comments and provide it for re-review. All reviewers agreed that experimental evolution is at the center of this study.

1. Please, consider and address carefully the critique of experimental evolution section by the first reviewer providing a more in-depth explanation of the choice of strain used in experimental evolution. In this context, it is important discuss in more detail other differences besides dim2 between the Zt09 and Zt10 strains.
2. Two of the reviewers comment on the origin and functionality of dim2 alleles that needs careful consideration.
3. The third reviewer raises an important issue of interpreting Zt09:DIM2 virulence phenotypes which also need closer attention.
4. Finally, provide more details on methodology and conclusions used throughout the study as outlined by reviewers.

**Reply:** We very much appreciate the positive feedback and thoughtful comments by the reviewers and editor, and believe that we have addressed all comments, including the four major items listed above.

**Reviewer #1:** The manuscript 'Recent loss of the Dim2 DNA methyltransferase decreases mutation rate in repeats and changes evolutionary trajectory in a fungal pathogen' by Möller and colleagues reports on computational and experimental analyses of the Dim2 DNA methyltransfereses in the fungal wheat pathogen Zymoseptoria tritici. The authors demonstrate that, next to conserved rid and dnmt5, few Z. tritici strains isolated at the centre of origin carry intact dim2 while many other isolates encode (multiple) inactivated versions of this gene. The authors furthermore show that dim2 is responsible for de novo DNA methylation (5mC) at transposable elements (TEs). Intriguingly, by performing experimental evolution experiments, the authors observed increased nucleotide changes C->T on CpA sites during mitosis. These mutations could contribute to the inactivity of TEs which therefore would shape the TE repertoires in strains polymorphic in dim2. This mutational processes seems to be analogous to the RIP genome defense mechanisms, which is RID dependent and thus far has been linked to the sexual cycle of fungi. This here described dim2-dependent mutator could therefore explain the occurrence of RIP-like mutations in many fungal genomes that lack apparent sexual cycles.

**I really enjoyed reading this paper. It is very clearly written, the data is presented in a very clear manner, and the results seem to support the authors' main conclusions. However, I am concerned about some aspects of the experimental evolution experiment the authors conducted to show that dim2 promotes C->T mutations during mitosis. It is unclear why the authors use a different isolate with non-functional dim2 (IPO323) compared with Zt09 that is used throughout the remainder of the manuscript. The authors indicated that Zt09 is derived from IPO32. Based on information I found in other papers from this group, Zt09 seems to be an isolate with a deletion of chromosome 18.**

**Thus, it is at least questionable to which extend the observations made on Zt09 throughout the manuscript can be easily transferred to IPO323 as it is not clear to which extent the genotypes differer (except del chr. 18, e.g. accumulation of other mutations throughout the genome).**

**Reply:** We appreciate the concern of the reviewer about the strains used in the evolution experiment. The evolution experiment was not initially designed to be a part of the Dim2 work, but an independent study conducted in the Stukenbrock lab aimed at comparing mutation rates between different isolates of *Z. tritici*; a separate manuscript by Habig *et al.* is now in preparation. It is correct that IPO323 and Zt09 differ in genotype, including the loss of chromosome 18 and a few additional mutations (described in Möller M, Habig M, Freitag M, Stukenbrock EH. Extraordinary Genome Instability and Widespread Chromosome Rearrangements During Vegetative Growth. Genetics. 2018 210: 517-529. doi:10.1534/genetics.118.301050). By the time of submission of this manuscript the SNP data for IPO323 and Zt10 were available, but we now have data that show there is no difference in mutation rate between IPO323 and Zt09 (IPO323Δchr18) indicating that genotypic differences between these two strains are not involved in accumulation of mutations. We have attached a figure here (Figure 1) that shows mutation rates in IPO323 and Zt09 (IPO323Δchr18). These data will be submitted as part of a manuscript in preparation.

**Figure 1:** Mutation rate of the two isolates IPO323 and IPO323Δchr18 (Zt09) assessed in a mutation accumulation experiment (Habig et al, in preparation). The *Z. tritici* isolates were propagated for 52 weeks on Yeast Malt Sucrose media at 18°C with weekly transfer of single cells (resembling a population bottleneck). Genomes of the evolved progeny strains were sequenced, and the accumulated mutations identified along the genomes.

**Furthermore, Zt09/IPO323 and Zt10 have been isolated from different regions and at different time points, and it is therefore conceivable that the genotypes of Zt10 and IPO323 differ not only in dim2. Consequently, the increased number of mutations in the evolved strains compared with the reference might therefore not only be due to differences in dim2.**

**To address these concerns, the authors need to corroborate their observations by performing the evolution experiments by using the deletion/complementation strains of Zt09 and Zt10 as here the genetic background is identical.**

**Reply:** This is an important point. Genotypes of different *Z. tritici* isolates vary greatly in terms of accessory chromosome complement, TE content and presence/absence of genes. This is also true for all 22 isolates we analyzed in this study. A recent paper published by Daniel Crolls group describes the genetic variation that is present in these isolates (Badet, T., Oggenfuss, U., Abraham, L. et al. A 19-isolate reference-quality global pangenome for the fungal wheat pathogen Zymoseptoria tritici. BMC Biol 18, 12 (2020). <https://doi.org/10.1186/s12915-020-0744-3>). Even though there is a lot of genetic variation between these isolates, we do find the specific genomic signatures reported here (reduced CA/TG and increased TA sites) in all isolates that contain a functional or recently inactivated *dim2* gene. These sites coincide with the target regions of Dim2 (repeats and CA sites that are only methylated in presence of a functional Dim2). Although it is possible that there are differences beyond *dim2* that influence the phenomenon we have described in our manuscript, based on our findings and analyses, presence/absence of a functional *dim2* is very likely to be the factor that promotes the differences in mutation rates.

We agree that ideally, we should compare mutation rates in isogenic deletion and integration strains. This is indeed an ongoing study dedicated to understanding the underlying mutation mechanism; clearly this is beyond the scope of a paper delineating the phenomenon. Because of COVID-19-dependent limitations for lab work during the past year on top of the duration of evolution experiments, we will not be able to include these experiments in this first manuscript about the role of Dim2 in promoting mutations.

**Some further detailed comments/suggestions:**

**L112: Could the authors please provide more detail how full-length dim2 was identified in Zt10?**

**Reply:** The reported loss of *dim2* in Z. tritici drew our attention to this genomic region. In previous comparative genome analyses of diverse *Zymoseptoria* species we discovered that some contained an intact *dim2*. This is now stated in the first section of the first paragraph.

**L221: The authors observe that introduction of dim2 into a dim2 deficient strain (Zt09) reduces virulence while deletion of dim2 in a dim2 competent strain (Zt10) does not show a corresponding phenotype (increase in virulence). Furthermore, the authors suggest (L485) that loss of dim2 is adaptive in an agricultural setting due to increased virulence. The authors really need to explain this observation in the first place and why increased DNA methylation would impact virulence.**

**Reply:** We agree with the reviewer that the impact of DNA methylation on virulence is likely limited but deserves further studies, yet these experiments go beyond the purpose and scope of the current investigation. Integration of *dim2* in Zt09 may have a negative impact on fitness since *dim2* has been lost for likely thousands of generations. The higher 5mC levels in Zt09::*dim2* compared to Zt10 indicate that there may be some adaptation to more balanced 5mC levels. Repeated re-infections with the Zt09::*dim2* strain may restore virulence to WT levels and reduce 5mC.

We added the following to our virulence paragraph:

“We note that restoring DNA methylation in Zt09 after absence of dim2 for likely thousands of generations does not necessarily reflect the influence that DNA methylation may have in Z. tritici under normal conditions. Deletion of dim2 in Zt10 did not have significant immediate impact on virulence; this needs to be further addressed in long term studies.”

We deleted our statement in the discussion saying that “increased 5mC levels negatively impact virulence”.

**L245: Could the authors provide a bit more detailed on the distribution of 5mC in the genome. How does the distribution of methylation levels look like (number of mC reads over C per position)?**

**Reply:** Thank you for this great suggestion. We added a new supplementary figure (S9) addressing 5mC levels and overlap of 5mC sites in the different strains and replicates. We found that methylation levels are very high in Zt09, where only few 5mC are maintained by Dnmt5, but lower in Zt10Δ*dim2*. In this strain, shortly after the loss of *dim2*, 5mC sites appear to be in the process of being lost with a high variability between replicates probably due to loss of 5mC sites by the expected incomplete maintenance of 5mC sites by Dnmt5. Zt10 and Zt09::*dim2* have comparable methylation levels and overlapping sites between replicates likely reflecting variation in 5mC levels in cell population in presence of a *de novo* methyltransferase. The reviewer’s suggestion resulted in new insights, thanks for this idea!

**L285: Obviously, this paper focuses on dim2 rather than on dnmt5. However, I feel the authors might have missed a huge opportunity by not providing additional functional data (e.g. deletion/complementation and WGBS) on the role of dnmt5. Recent work by Bewick and colleagues demonstrated that the majority of fungi encode dim2, dnmt5, and rid (Bewick et al. 2019). However, most work to my knowledge on DNA methylation in fungi has focused on systems with only two DNA methyltransferases (dim2 and rid, e.g. Neurospora). Studying a system with all three enzymes would provide an intriguing opportunity.**

**Reply:** We agree that the analysis of all three DNMTs would be very interesting. A recent paper from Bart Thomma’s group (Kramer et al., 2020; <https://doi.org/10.1101/2020.08.26.268789>) analyzes all three DNMTs in *Verticillium dahliae*. Based on their data and previous studies in *Cryptococcus* and *Neurospora* we believe that the role of the other two methyltransferases is probably very limited in terms of 5mC levels. Nevertheless, in future studies we want to investigate if these two DNMTs may play a role in mediating the CA->TA mutations we describe in this study.

**L297: How do the authors explain the grouping of UR95 in the correspondence analyses?**

**Reply:** This is a very good point that we now address in more detail in the results section. UR95 is different from the other “recently inactivated” strains as it contains two *dim2* copies with very few mutations (one native, one additional) and the remaining additional copies show relatively high sequence identity (comparable to the additional copies in the “non-functional” category). Both, the high sequence similarity of the additional copies and the fact that there are two “recently inactivated” *dim2* genes increases the likelihood of rapid mutation and inactivation. Based on these observations and the grouping in the correspondence analysis we believe that *dim2* was only active for a very short time (or not at all) in this isolate. Rather than speculate in this paper, we will address this issue by testing such a situation in our ongoing studies by use of isogenic strains.

**L397: This is similar to the point raised for Fig 2. The authors seem to infer that dim2 alleles with polymorphisms except premature stop codons are functionally active. First, do this polymorphisms alter amino-acid residues, for instance in the catalytic domain. Second, to ascertain that these alleles are functional, the authors need to provide additional data that these alleles are indeed functional.**

**Reply:** Based on the comments of reviewer 1 and 2 we show our analysis of amino acid sequence between Dim2 alleles in detail (S3 Fig and results). All presumably or known functional Dim2 alleles (e.g., from Zt10) vary in minor. This is not an uncommon observation for *Z. tritici*, where a high number of SNPs between different isolates has been described in several studies; the same is also true for alleles obtained from five different strains of *N. crassa*. As shown in figure S3B, missense mutations are not located in any known functionally important regions of the protein. For example, except for the “transversion allele”, the majority of non-synonymous SNPs are located outside of the DNA methyltransferase domain. The alleles that show the greatest variance are those with the 5’ deletion. Here, we found additional InDels and a short C-terminal extension of the protein (9 aa), but again, the deletions are not in regions that are conserved in Dim2 proteins when comparing all fungi. All of the InDels maintain the reading frame. This 5’ deletion allele is found in two of the presumed functional Dim2 strains and three recently inactivated strains. It is not unusual to find several alleles for the same gene in diverse populations of the same species, and we think that this is the case here; based on the data now presented in figure S3B we propose that there are at least three different functional *dim2* alleles in extant *Z. tritici* populations. We agree that additional data we will obtain in the future by allele swaps will be helpful but based on the current sequence analysis and comparisons with Dim2 from other ascomycetes with Dim2-dependent 5mC methylation we are convinced that these alleles are functional.

**L467: Could the authors provide more details how the different DNA methlytransferases are expressed in Z. tritici? Is dim2, for example, constitutively expressed?**

**Reply:** This is a good suggestion. We added the expression profiles over the time course of infection and *in vitro* (Zt09 only) for Zt09 and Zt10 as supplementary figure S10. The expression of *rid*, which is still not known to be a methyltransferase in any organism, even though it has a DNMT domain, is very low in both isolates, as expected, since *rid* is expressed pre-meiotically. *dim2* (data for Zt10) is expressed at low, but constant levels *in vitro* and during infection. The expression of *dnmt5* varies during infection and peaks during the switch from biotrophic to necrotrophic growth. This is consistent between the two isolates, whereas the expression level of *dnmt5* in Zt10 is overall higher compared to Zt09.

**L503: Could the authors please provide some brief details how genome assemblies were performed?**

**Reply:** We now added details about the software used for generating and determining the quality of the assemblies. A more detailed description is available in the referenced publication (Feurtey et al., 2020).

**L530: Was the search with the DNA methyltransferase domain done using BLAST or via profile search against the genome?**

**Reply:** We used BLAST against the *Z. tritici* genome and added this information in the methods part.

**Fig. 2: The authors identify multiple dim2 that contain an 5' deletion. Nevertheless, they conclude that some of these might be intact, thus active. For example, Zt289 contain this deletion. However the authors do not provide any evidence that this allele is indeed functional. I would suggest to at least briefly address this possible ambiguity in the results/discussion.**

**Reply:** See response to comment above.

**Fig. 3: Can the authors please provide an explanation why the number of methylated sites is higher in the Zt09::dim2 strain compared with Zt10? is there a difference in copy-number of expression of the dim2 allele between the isolates?**

**Reply:** This is a very interesting question. We do not have expression data for the Zt09::*dim2* strain but based on integration in the native locus and no additional integration events (based on Southern blot) we would expect that expression levels are similar between Zt10 and Zt09::*dim2*. It is possible that upon integration of *dim2*, a high number of sites is methylated that is then progressively reduced after several rounds of infection or prolonged growth. Another possibility is that DNA methylation levels vary between different isolates based on TE content and frequency of specific TE families that may attract higher methylation levels. We have added these hypotheses about differences in methylated sites to the results section.

**Fig. 4: For clarity, could the authors please indicate in panel a and b the different dim2 classes (active, inactive, etc.) based on e.g. differences in color or shapes?**

**Reply:** We agree that this would be very helpful. We have tried but found that the R package used to generate the correspondence analysis and the resulting plot does, to our best knowledge, not allow these parameters to be changed.

**Reviewer #2:** Although a wheat pathogen, Zymoseptoria tritici is a tractable lab model to study fungal genome evolution. In this manuscript, Möller et al. present the identification of Z. tritici isolates carrying a functional dim2 gene while strains of reference display inactivated dim2 genes and low levels of 5mC. They showed that presence of functional dim2 alleles, either from endogenous or transgenic origin, correlates with significant levels of 5mC in TEs. Importantly, an evolution experiment conducted over 1000 mitotic divisions reveals that functional dim2 enzyme alters nucleotide composition of TE, in a RIP independent manner. This finding is a real breakthrough in the field of fungal genome evolution. Overall, the quality of this manuscript is suitable for publication in PLOS Genetics, providing some modifications and clarifications that are listed below.

**The major concern deals with the mutation patterns of the de-RIPed non-native dim2 alleles (Figure 2), which strongly suggest a clonal origin of all of the sequenced subtelomeric copies. Consequently, the authors’ statement that “all non-native alleles share a specific pattern of transition and, surprisingly, transversion mutations in the DNA methyltransferase domain” does not fully fit with these data. More than specific, these are almost identical patterns, likely inherited from a single integration event of the so-called ‘transversion allele’ in an ancestral strain lacking dim2 activity. The text should be modified adequately. The authors should also consider using the de-RIPed dim2 phylogenetic analysis present in this study (Figure S1 A) to address questions linked to the clonal origin of the ‘transversion allele’, namely when and where (i.e. in which strain) this integration event might have happened.**

**Reply:** We agree with the reviewer that this pattern of mutations in the transversion allele indicates a clonal origin. This was not stated explicitly enough in our manuscript, so we have added the following sentence to make this clearer:

“The presence of the same transversion mutations in all additional copies indicates that this allele may have resulted from a single integration event in an ancestral *Z. tritici* isolate.” We would like to avoid using the term “clonal origin” because we don’t actually know that there was a single event, though we suspect that this was the case.

It would be very interesting to investigate the origin of the transversion allele. Based on our findings that functional alleles of the transversion allele may be present in the population and repeated integration of these alleles may change dynamics of sequence degeneration it will be difficult to determine the evolutionary history of these *dim2* alleles. Additionally, the de-RIP approach affects all mutations that look like they may originate from RIP, potentially artificially reducing the amount of actual variation.

**The “native dim2 gene” expression is misleading since “native” would mean “functional” is most readers’ mind, whereas here, it is used to qualify a genomic location rather than a functional feature. So I would rather use “resident dim2 gene” or alternatively “dim2 native locus”.**

**Reply:** We thank the reviewer for this comment and agree that this may be misleading to some readers. We thought about the proper terminology at great length. We thought about using ‘endogenous’ *vs.* ‘ectopic’. Similarly, ‘wild-type’ and ‘mutant’ were rejected because in this study several alleles are ‘wild-type’. A ‘native’ gene may still have non-functional alleles. Consequently, we tried to be as specific as possible throughout the whole manuscript. We therefore also clarified our definition of ‘native’ and ‘functional’ in this manuscript.

“Throughout this study, we refer to *dim2* in the original locus on chromosome 6, functional or non-functional, as the ‘native’ gene or allele; *dim2* copies outside the native locus are referred to as ‘non-native’ or ‘additional’ alleles.”

**- Abstract: “Integration of a functional dim2 allele in strains with mutated dim2 restored normal 5mC levels, demonstrating de novo cytosine methylation activity of dim2 this is an overstatement”.**

**The authors did not demonstrate de novo methylation activity of dim2 and alternative possibilities exist. For one, 5mC patterns could very well be installed by non-DIM2 DNMT(s) and further maintained by DIM2.**

**Reply:** Dim2 has been described as a *de novo* methyltransferase in *Neurospora* and our findings, restoring high levels of 5mC and non-CpG methylation, clearly indicate that Dim2 has *de novo* methylation activity. There is no indication that either of the other two DNA methyltransferases, Dnmt5 or Rid, have any *de novo* activity. We do not think that it is an overstatement to attribute Dim2 *de novo* methylation activity since it aligns with previously published findings and the data presented in our study.

**- line 120: “Five Z. tritici isolates contained an intact, non-mutated copy of dim2 in addition to multiple mutated, non-functional copies”.**

**How is it possible that an intact non-mutated copy of dim2 persists, meaning that it escaped RIP, when multiple mutated, non-functional copies are also present in the same haploid genome? Are they some data related to RIP efficiency on repeats located in cis vs in trans in Z. tritici’s genome? Alternatively, would you postulate that these strains did not reproduce sexually?**

**Reply:** This is indeed a very interesting point that we have also been considering. As discussed in the manuscript, RIP needs a certain degree of homology to efficiently induce mutations (for a review, see Galagan JE, Selker EU. RIP: The evolutionary cost of genome defense. Trends Genet.; 2004;20: 417–423. doi:10.1016/j.tig.2004.07.007). We only find functional *dim2* genes when there are either no additional copies (Zt469), or the additional copies are more diverged from the functional *dim2* (Fig 2B). There are currently no studies on RIP efficiency in *Z. tritici* but based on the very clear pattern in Fig 2B, we postulate that the functional *dim2* genes are not, or only at a very low rate, targeted by RIP when the sequence identity of the additional copies falls under a certain threshold. RIP is not 100% efficient, especially if duplications are unlinked, and there are conditions under which progeny have only few, or no mutations (e.g., early spores in *N. crassa*) so that non-mutated progeny can be obtained from any cross and those may be selected upon. However, even reduced RIP efficiency may be enough to induce mutations over time that eventually result in inactivation. This would be one explanation for the presence of recently inactivated alleles we found in some isolates.

**- p148: Any polymorphism among functional Dim2 alleles of the strains from Iran? On a more general note, it would be interesting to give more evolutionary depth to this descriptive paragraph by performing dN/dS analyses, at least for conserved transversion mutations within the catalytic domain, eventually compared to the few ones outside of it.**

**Reply:** We have added additional figure S3 and sentences in the results section to point out the differences in protein sequences of functional Dim2 alleles. We find variation in almost all isolates and most variation in the alleles that contain a 5’ deletion. None of these mutations affect known functionally important sites. Almost none of these non-synonymous mutations in the functional alleles seem to be conserved between different isolates except for the mutations in the DNA methyltransferase domain in the ‘transversion allele’.

**- line 157: Are they from a specific retrotransposon family?**

**Reply:** Yes, most additional *dim2* copies have transposons that are identified as RIX (LINE) elements in their flanking regions. We have added this information in the results part.

“The TEs that are found in the flanking regions of most of the additional *dim2* copies belong to the LINE family.”

**- line 182: “This second pattern was unexpected; the most parsimonious explanation is replacement of non-functional alleles at the native locus with novel dim2 alleles, perhaps by recombination with wild grass infecting isolates, such as Zt469.”**

**On what ground? The authors should underpin this hypothesis with further mechanistic explanation. Is there any established example of such a replacement to refer to? Besides, the authors stated in the discussion section that “Further analyses of diverse Z. tritici isolates from different hosts and geographical locations (T. aestivum , Triticum durum , or Aegilops sp.) are needed to track the evolutionary history of the dim2 gene in the Zymoseptoria species complex. ”Therefore if it is not known for sure that wheat-pathogen strains and wild-grass infecting strains are present at the locations (Table S2), how to argue for inter-specific recombination events?**

**Reply:** We agree that this statement is speculative and the way we phrased the sentence indicates that this is one of our hypotheses. There is evidence for introgression between different species of the *Zymoseptoria* species complex (Alice Feurtey, Danielle M Stevens, Wolfgang Stephan, Eva H Stukenbrock, Interspecific Gene Exchange Introduces High Genetic Variability in Crop Pathogen, Genome Biology and Evolution, <https://doi.org/10.1093/gbe/evz224>). So far, all wild-grass infecting isolates have been collected in Iran and the majority of the *Z. tritici* strains that contain a functional *dim2* were collected in Iran as well. We argue that this is a possible explanation of how functional *dim2* alleles can be acquired, and added the following sentence to this section to support our hypothesis:

“Introgression events between *Zymoseptoria* species have recently been described and wild grass infecting *Z. tritici* isolates and sister species and wheat infecting *Z. tritici* populations co-exist in the same geographical regions in the Middle East.”

**- Figure 2A: It would be handy to give some structural information about end/start of the dim2 CDS and location of conserved N-term motifs of the dim2 enzyme.**

**Reply:** This is a very good suggestion. In addition to the DNA methyltransferase domain, there are two BAH domains annotated. There are no predicted domains close to the N-terminus. We added a schematic of Dim2 including the predicted domains to Fig 2A and supplied complete protein sequences with all known conserved and a few newly discovered conserved regions in figure S3B.

**- line 229 and Fig S6: in order to properly demonstrated that fragments of higher molecular weight generated by 5mC-sensitive enzymes are readily due to DNA methylation and not to partial enzymatic reactions, these blots have to be hybridized using a single unmethylted gene as a probe.**

**Reply:** We agree with the reviewer that this would be necessary to rule out partial enzymatic reactions. We tried to make the reactions as comparable as possible by using equal amounts of DNA and enzyme for each reaction but as the reviewer pointed out correctly this does not guarantee complete restriction. However, since we used these Southern blots as an additional confirmation for the presence/absence of 5mC in addition to two independent bisulfite conversion reactions of three replicates per strain followed by either Sanger sequencing of target loci or whole genome bisulfite sequencing, we are confident that we were able to detect differences in 5mC methylation in these strains.

**- line 255: “a de novo DNA methyltransferase is present.”**

**Which one are you referring to?**

**Reply:** We are referring to the only known *de novo* methyltransferase in fungi, Dim2. Dnmt5 is considered a maintenance DMT, and Rid has no known DMT activity, although its homolog in *Ascobolus* was shown to cause *de novo* methylation in the sexual cycle only. We added Dim2 to this sentence to clarify this.

**- lines 267-286: What is the point of this paragraph? This is more of general background and should be moved either in S8 figure legend for the first part or in discussion section for the last sentences.**

**Reply:** We think this paragraph is important because it offers an explanation for the presence of DNA methylation even in absence of *dim2*. We furthermore added a new supplementary figure for this paragraph showing the expression patterns of the three (putative) DNA methyltransferases throughout the infection cycle of *Z. tritici*, as requested by reviewer 1.

**-lines 415-416: “A likely scenario for repeated invasion and inactivation cycles predicts that some wild grass infecting isolates carried “transversion alleles” that were not mutated.”**

**If the ‘tranversion alleles’ are the non-native mutated ones showed fig2A, this sentence is puzzling.**

**Reply:** We agree that this sentence may sound puzzling. In this paragraph, we are discussing the origin of the non-native *dim2* allele that has transversion mutations in the DNA methyltransferase domain. We found evidence that there are also potentially functional alleles of the transversion allele in other populations (Australian isolate mentioned in the discussion, allele in the Ukrainian isolate that has very few mutations) and based on protein sequence comparisons, this allele is quite similar to the current native *Z. tritici* alleles, much more so than *dim2* of any of the sister species. We therefore propose that there are (or have been) different *dim2* alleles in the native locus, one of them the transversion allele whose amplification led to inactivation of *dim2*. So far, we did not find a functional transversion allele in the native locus, but two isolates (CRI10 and OregS90) have a non-functional transversion allele in the native locus. The presence of putative functional transversion alleles suggest that there may be isolates, possibly wild-grass-infecting isolates, that still carry a functional transversion allele in the native locus or elsewhere in the genome. This could lead to reacquisition of a functional allele or inactivation (high sequence similarity to functional *dim2* followed by RIP).

**- lines 482-484: “Here we uncovered a system of continuing loss and re-acquisition of the ability to silence or inactivate TEs in the genomes of numerous isolates of an important plant pathogen, Z. tritici.”**

**Still speculative, this sentence should be rephrased.**

**Reply:** We have rephrased this sentence.

“Here we uncovered a system that suggests continuing loss and re-acquisition of the ability to silence or inactivate TEs in the genomes of numerous isolates of an important plant pathogen, *Z. tritici*.”

**Reviewer #3: I would like to congratulate the authors on a great manuscript that I already enjoyed reading the preprint. I also would like to apologize for being really late with the reviews.**

**The manuscript describes an evolutionary study of the DNA methyltransferase DIM2 in the important plant pathogen Z. tritici. The study is very timely and very well executed. I only have a couple of minor suggestions and comments that are easily addressed by re-writing.**

**Minor points:**

*** p. 6 140ff and Figure 2. It would be great if the text and the figure could be linked some more. Right now this is a bit difficult to follow. For example, l 179 mentions pattern 2 and this is not explained in the figure. I think the authors talk about three patterns. This should be more explicit in the text and in the figure.**

**Reply:** Thank you for pointing this out, the text and figure were indeed not well connected. We now mention every pattern more explicitly in the text by linking it to the color code and edited the figure so that the patterns are listed in 2B.

*** I am a bit confused by the usage of dim2 as active and inactive. The common conventions followed is that functional alleles are all capital italic "DIM2", non-functional low case italic dim2, and protein variants as all capital DIM2. This is potentially something the authors want to take on board.**

**Reply:** In this manuscript we followed the nomenclature used by others for *Z. tritici.* The reviewer suggests nomenclature as used in budding yeast. Here, Dim2 means the protein and *dim2* means the gene. By itself this does not state what allele is found at the locus and we specify this for each case in the manuscript. We replaced ‚active’ and ‚inactive’ by ‚functional’ and ‚non-functional’ or ‚mutated’ as required.

*** The authors state that methylation has a negative effect on infection in wheat based on Fig. S5 where a functional DIM2 is transformed into Zt09 (and potentially that Zt10 having a functional allele is not as infective). What is the sample size of transformants tested here? Could this be a pleitropic effect of the gene insertion? How does the mutation of Zt09:DIM2 alter this phenotype? There is no need to do new experiments if these are too long-winded. Yet I would encourage the authors to consider these alternative explanations for the observed reduced virulence in this transgenic Z. tritici line.**

**Reply:** This is a valid point that was also addressed by reviewer 1. Based on our data (the infection data come from two independent infection experiments including the mutants; two independent transformants and respective WT strains, 35 infected leaves per strain/experiment) we think that the effect of DNA methylation on virulence under these conditions is limited. Integration or deletion of *dim2* followed by growth *in vitro* before infection does likely not reflect the actual fitness differences, if there are any, of isolates in the field that have adapted to low or high 5mC levels. We have modified the text accordingly, see response to reviewer 1.

*** The insight gained by the correspondence analysis wasn't immediate obvious to me. I am also not familiar with this analysis. This could be explained a bit more or supported with additional analysis e.g. what are the relative abundance of CG or GA sides in +dim2 lines TEs?**

**Reply:** We provide supplementary table S3 that lists relative abundances of all dinucleotides in the genomes of all isolates used in this study. We calculated frequencies for both, TEs and TE-masked genomes and found that relative abundances of CA/TG and TA sites are different in TEs but not in the masked genomes. Correspondence analysis was then used to visualize the relationships of dinucleotide frequencies and strains. We rephrased the sentence that mentions the correspondence analysis to: “Therefore, we extracted TE sequences, calculated dinucleotide frequencies relative to the TE content for each genome (S3 Table), and visualized the difference in relative frequencies of specific dinucleotides in TEs in the different isolates by correspondence analysis.”

We hope this sentence and table S3 clarify the analysis of dinucleotide frequencies.

*** The method section could include version numbers for all programs used with specific flags if any were evoked.**

**Reply:** We have updated the methods section to include software and version used and included a supplementary file that lists all programs, version numbers and commands for each step of the analysis.

**I really enjoyed the evolution experiment. Nicely done.**

**I don't have anything to add to the discussion as my suggestion were already included based on the comments on the preprint.**
